# Effects of Fusarium Head Blight on Wheat Grain and Malt Infected by *Fusarium culmorum*

**DOI:** 10.3390/toxins10010017

**Published:** 2017-12-27

**Authors:** Valentina Spanic, Tihana Marcek, Ivan Abicic, Bojan Sarkanj

**Affiliations:** 1 Department of Small Cereal Crops, Agricultural Institute Osijek, Juzno Predgradje 17, HR-31000 Osijek, Croatia; ivan.abicic@poljinos.hr; 2 Faculty of Food Technology Osijek, Josip Juraj Strossmayer University of Osijek, Franje Kuhača 20, HR-31000 Osijek, Croatia; tihana.marcek@ptfos.hr (T.M.); bojan.sarkanj@ptfos.hr (B.S.)

**Keywords:** Fusarium, grain, malt, mycotoxins, wheat

## Abstract

*Fusarium* head blight is a destructive disease of cereals worldwide. The aim of this research was to study the effect of heavy Fusarium infection with *Fusarium culmorum* and biosynthesis of mycotoxins on different wheat varieties during malting by setting up field trials with control and *Fusarium*-inoculated treatments at the Agricultural Institute Osijek. The highest occurrence of *Fusarium* mycotoxins was expectedly recorded in susceptible variety in grain and malt (3247 and 1484 µg kg^−1^ for deoxynivalenol (DON), 735 and 1116 µg kg^−1^ for 3-acetyl deoxynivalenol (3-ADON), 37 and 233 µg kg^−1^ for zearalenone (ZEN), respectively). Based on published information, complemented by our own results, the following conclusions can be drawn: The presence of 3-ADON in different wheat varieties might be the result of its conversion into DON by deacetylation during the malting process. The detection of the mycotoxin ZEN indicated that this mycotoxin is only specific for wheat malt.

## 1. Introduction

More than 200 *Fusarium* species are known, but the most dominant in wheat are *Fusarium graminearum* Schwabe (teleomorph Gibberella zeae), *F. culmorum* (Wm. G. Sm.) Sacc. (teleomorph unknown) and *F. avenaceum* (Fr.:Fr.) Sacc. (teleomorph *Gibberella avenacea*) [1]. Although the disease causes a reduction in the yield and quality of wheat, a major concern in food and feed is the contamination of grain infected with fungal secondary metabolites (mycotoxins), such as trichotecenes [2], zearelenone (ZEN) and moniliformin (MON), which may pose health risks to humans and animals [3]. Also, *Fusarium* head blight (FHB) causes sterility, poor grain filling and test weight decrease thus resulting in significant yield loss. Currently, agronomic practice and fungicides may only partially reduce the risk of damage. In some studies, fungicide treatment reduced the infection by up to 15% [4], and some researchers have reported reductions of even up to 50% [5]. The effectiveness of fungicide treatment depends on various factors such as: contamination level (both *Fusarium* and co-contamination with other fungal species), health status of the plant (oxidative stress of the plant, resistance of the wheat genotype), drought level, concentration and mode of action of fungicide etc. The best approach to control FHB and to reduce mycotoxin contamination is to create wheat genotypes that carry effective resistance genes [6,7,8]. The resistance reaction of wheat to Fusarium infection includes the following components: Type I, resistance to initial infection [9]; Type II, resistance to spread of symptoms [9]; Type III, resistance to toxin accumulation [10]; Type IV, resistance to kernel infection [11,12]; Type V, yield tolerance [11,12].

The occurrence, amount, and type of mycotoxins can depend on weather conditions [13,14], geographic location, fungi species, infection severity and variety resistance. *F. graminearum* and *F. culmorum* are the most important DON producers, and belong to trichothecenes B group, which are characterized as inhibitors of protein synthesis and effectors of the immune system [15]. Other *Fusarium* species that cause wheat disease include *F. avenaceum* (Fr.) Sacc. and *F. poae* (Peck) Wollenw. Those species are good producers of moniliformin (MON), beauvericin (BEA) and enniatins (ENNs) [16], and their presence has been observed in wheat before [17].

DON, nivalenol (NIV), T-2 toxin (T-2), HT-2 toxin (HT-2), ZEN and the fumonisins B (FB) are known as “field mycotoxins” [18]. They occur under favorable conditions such as high moisture and temperature during the flowering of wheat, but they can also increase as a result of improper storage or processing [19,20]. European Commission regulation (EU) 1881/2006 set limits at 1250 µg kg^−1^ for DON in unprocessed cereals other than durum wheat, oats and maize and 100 µg kg^−1^ for ZEN in unprocessed cereals [21]. Maximum levels for sum of T-2 and HT-2 of up to 100 µg kg^−1^ for wheat, rye and other cereals have been recommended by European Commission recommendation (EU 165/2013). The concentration of DON in barley malt has not yet been regulated, but it could be a potential problem. Along with food safety issues due to mycotoxins, the *Fusarium* infections also affect the quality of wort and beer [22]. Enzymes responsible for starch decomposition during malting process are activated thus creating favourable conditions for the rapid growth and development of bacteria, yeast and fungi naturally present in cereals [23]. In general, food processes with high temperatures can reduce mycotoxin concentrations significantly, but cannot eliminate them completely [24]. DON is stable up to 170 °C, and is an inhibitor of protein synthesis. It affects germination and other metabolic processes in the germinating grain and modulates the activity of proteolytic enzymes and gibberellins, which are promotors of enzyme synthesis in the barley [25]. In addition, *Fusarium* infection may be associated with “gushing” in the resultant beer [25]. Moreover, significant correlations exist between the intensity of gushing and levels of DON in barley and malt, ergosterol in malt, and ZEN in malt [26]. In this study, the effects of *F. culmorum* on the production of different mycotoxins in the grain and corresponding malts after artificial inoculation of winter wheat varieties in the field were investigated. The present study also provides evidence and new knowledge on the impacts of the FHB pathogen complex on the malting of naturally and artificially infected wheat, because most previous research was focused on barley malt.

## 2. Results

**Figure 1:**
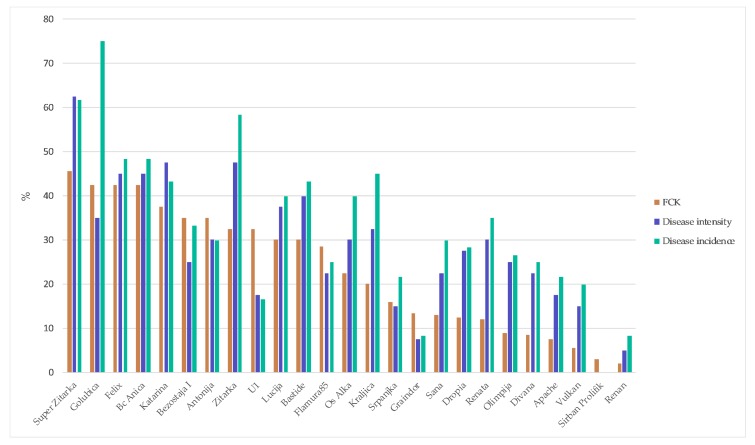
Disease incidence and intensity, *Fusarium*-colonized kernels (FCK) for 25 wheat varieties artificially inoculated with *F. culmorum*.

Disease incidence of contamination ranged from 0 (‘Sirban Prolifik’) to 75% (‘Golubica’). General resistance (disease intensity) ranged from 0 (‘Sirban Prolifik’) to 63% (‘Super Zitarka’). Changes in FCK were less marked, with FCK from 2% (‘Renan’) to 46% (‘Super Zitarka’) (Figure 1).

**Figure 2:**
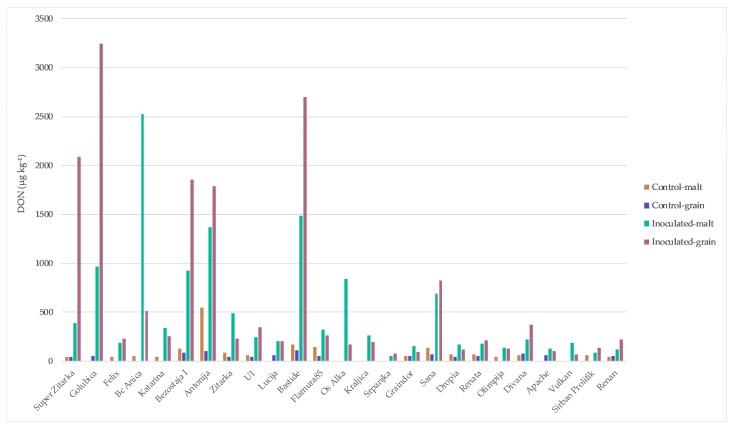
Results of DON analysis for 25 winter wheat varieties in the grain and malt in control treatment and after spray inoculation with *F. culmorum* during flowering stage.

**Figure 3:**
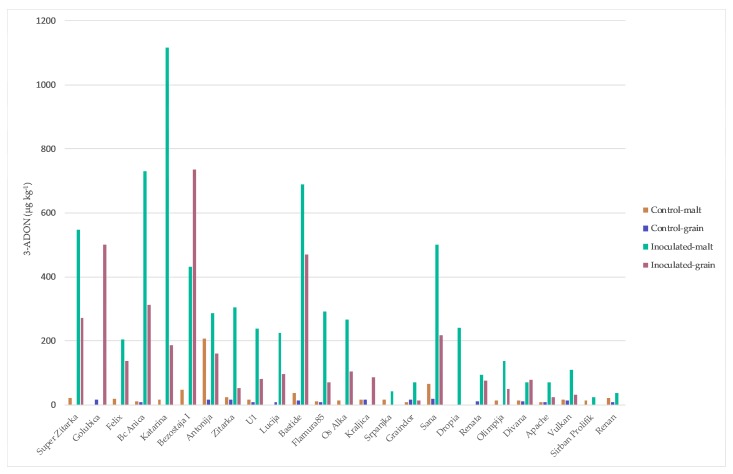
Results of 3-ADON analysis for 25 winter wheat varieties in the grain and malt in control treatment and after spray inoculation with *F. culmorum* during flowering stage.

**Figure 4:**
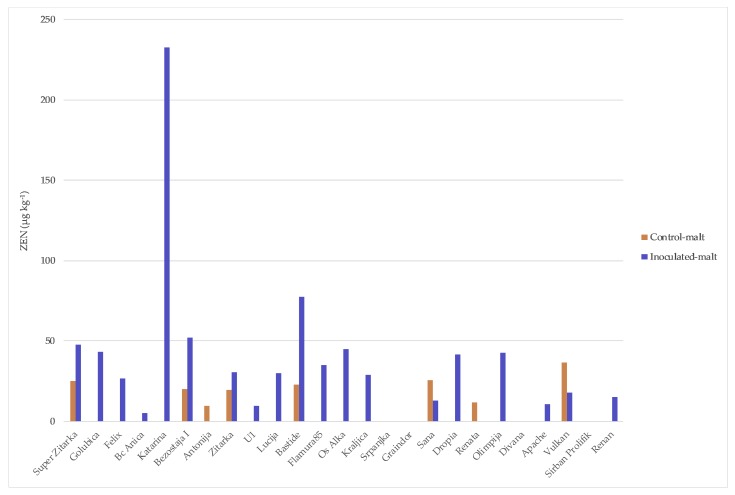
Results of ZEN analysis for 25 winter wheat varieties in the grain and malt in control treatment and after spray inoculation with *F. culmorum* during flowering stage.

Mycotoxin concentrations in wheat grain and malt determined in 2014/2015 are summarized in Figure 2, Figure 3 and Figure 4. Naturally infected grains (control) exhibited a high percentage of positive samples for DON and 3-ADON (64%) (Figure 2 and Figure 3) and for T-2 and HT-2 sum (16%) (data not shown). DON in control grains ranged from 0 to 115.62 µg kg^−1^, while 3-ADON was much lower, ranging from 0 to 17.86 µg kg^−1^. In contrast, the highest level of the DON occurrence was recorded in inoculated grain and malt (100% of total samples) (68.55–3246.53 and 51.18–2526.30 µg kg^−1^, respectively) (Figure 2) and for 3-ADON in inoculated grain (84% of total samples) (13.42–735.38 µg kg^−1^) and in the corresponding malt, it increased (92% of total samples) (25.07–1115.60 µg kg^−1^) (Figure 3). The highest percentage of ZEN-positive samples was recorded in inoculum malt, 76% versus 32% in control malt, within range of 5.12–232.57 µg kg^−1^ and 9.34–36.92 µg kg^−1^, respectively (Figure 4). Some samples did not show the DON contamination both in control grain and malt (‘Srpanjka’, ‘Kraljica’, ‘Vulkan’ and ‘Os Alka’). Figure 2 indicates that the most naturally DON-contaminated grain (control) was the variety ‘Bastide’ (116 µg kg^−1^), and, for the malt variety, ‘Antonija’ (548 µg kg^−1^). The presence of DON in the control grain was undetected in only nine of 25 samples. Five DON values measured in inoculated grains that year (1791, 1857, 2091, 2698 and 3247 µg kg^−1^) (‘Antonija’, ‘Bezostaja I’, ‘Super Zitarka’, ‘Bastide’ and ‘Golubica’), and three DON values measured in inoculated malt (1367, 1484 and 2526 µg kg^−1^) (‘Antonija’, ‘Bastide’ and ‘Bc Anica’), exceeded the maximum allowable limit for unprocessed cereals of 1250 µg kg^−1^ set by the European Union (EC 1881/2006). Correlation analyses of the complete set of 25 winter wheat varieties showed a significant positive relation for DON contamination between control and inoculated grain (0.56) and between control grain and control malt (0.59), as well as between DON contaminations in inoculated grain and malt (0.52) (Table 1). There was not any significant correlation for control and inoculated malt (data not shown). Actual 3-ADON concentrations were generally lower than DON concentrations, with mean levels ranging from <0.1 (the limit of detection) to 18 and 208 µg kg^−1^ in control grain and malt, respectively. The highest concentrations in inoculum grain and malt were 735 and 1116 µg kg^−1^ (Figure 3). There were no significant correlations in 3-ADON concentration between samples in inoculum grain and malt or control grain and malt (data not shown). ZEN concentration was analyzed in both grain and malt samples. Only one sample in inoculum malt exceeded the legal limit at 100 µg kg^−1^ for feed cereals in the EU (‘Katarina’, 233 µg kg^−1^) (Figure 4). No significant correlation was obtained for control and inoculum malt for ZEN concentrations (data not shown). Incidence of T-2 and HT-2 toxins was low; only four of 25 samples of control grain contained detectable levels, and two of 25 samples of inoculated grain were positive on ZEN with losses during malting (data not shown).

## 3. Discussion

DON and 3-ADON were the most-occurring mycotoxins in the control wheat samples, while ZEN was detected in both control and infected malt samples. It was expected that mycotoxin contents in grain samples would vary in different winter wheat varieties of inoculated treatment, even though plots were artificially inoculated with spores of *F. culmorum*. The infection rates of non-inoculated plots were 0, but they varied in inoculated plots up to 63% for general *Fusarium* resistance. The susceptible varieties, such as ‘Golubica’, ‘Super Zitarka’ and ‘BC Anica’, were characterized by a much greater accumulation of DON than the resistant varieties. Moderate statistically significant correlations were observed between DON in control and inoculated grain and between control grain and control malt, as well as between DON contaminations in inoculated grain and malt. This relationship suggests that samples with high DON levels would likely produce malt with high DON levels, so resistance of the crop should be a major safety factor for the beer production. This was also concluded by Schwarz et al. (2006) [27] for barley samples; however, due to large amount of unexplained variation, they concluded that this relationship needs to be further investigated in details. The same was noticed by Váňová et al. (2004) [28], who revealed that content of DON increased in most cases after the barley samples were malted. Moreover, Schapira et al. (1989) [29] concluded that T-2 toxin, DAS and DON had the potential to affect malting and malt characteristics, with the possibility that even at the much lower natural levels in grains, these mycotoxins acting additively or synergistically with others may have a deleterious effect on malting. The present study also provides evidence of new knowledge on the impacts of the FHB pathogen complex on the malting of naturally and artificially infected wheat, because most of previous research has been focused on barley malt. The focus of this research with wheat-infected malting was to explore the effect of *Fusarium* sp. on wheat malt; namely, because during malting, this process seems to represent a critical step within its temperature range, and mycotoxin production can be six times higher than under optimal temperatures, which consequently promotes higher fungus growth [30]. During the brewing process, significant increases in the levels of mycotoxins can occur [31]. Previously, *F. graminearum*, *F. culmorum* and *F. poae* have been named as active gushing inducers that cause quick uncontrolled spontaneous over-foaming immediately after a bottle or can opening [25]. In further research, we will check stability of mycotoxin occurrence in different environments.

DON concentrations for barley were, however, significantly lower than was reported by Lancova et al. (2008) [31]. In our research, there was also a corresponding increase in DON concentrations, from a mean of 41 µg kg^−1^ for control grains to 75 µg kg^−1^ for control malt. Maximum concentrations in inoculum treatment were greater than 1500 µg kg^−1^ in both grain and malt in a few samples, although the EU limit of 1250 µg kg^−1^ for wheat was not exceeded for and of the 25 samples. There was a statistically significant correlation between DON and 3-ADON concentrations, except in control grain. In general, malt samples that were artificially inoculated contained less DON than the samples from which they were prepared, indicating that toxin was lost or transformed during the commercial malting process. There were 9 of 25 samples in which the malt had more DON than the grain. In the control treatment, 14 of 25 samples had a higher DON concentration after malting. Malting wheat samples showed somewhat different picture for control and inoculated treatment, where in the control and inoculated treatment, only 4 of the 25 samples had less 3-ADON after malting. Our results are not in accordance with Váňová et al. (2004) [28], who revealed that DON was not detected in barley malt in the non-treated control, but DON concentration depended on the specific climate of the testing year and position of the test field. According to Schwarz et al. (1995, 1997) [32,33], production of mycotoxins during malting is difficult to predict from the original barley, and is probably dependent upon viability, as well as the original level of infection. In addition to that, Wolf-Hall and Schwarz (2002) [21] stated that during the steeping process, the level of DON can sometimes be decreased, and is no longer detectable. Similar findings were observed for the barley malt and barley grains, which showed similar lower level of DON [25]. Contrarily, in the research of Lancova et al. (2008) [31], in malt, the content of monitored mycotoxins was higher compared with the original barley (grains without malting). In the relatively wet season of 2014/2015 occasional malt samples had a higher concentration levels of DON or 3-ADON than the starting wheat grain (control), but this was not the case with T-2 or HT-2, which are two of the most toxic members of type-A trichothecenes, produced by a number of *Fusarium* species. According to EC 165/2013, these positive samples were only dangerous for cereal-based foods for infants and young children (>15 µg kg^−1^ Following mycotoxins, mainly NIV, (diacetoksiscirpenol) DAS and/or fusarenon X (FUS X) have not been found in control and inoculated samples.

Mean and maximum values of mycotoxins were lower in the control treatment, both for grain and malt, and no samples exceeded legal limits for human consumption. In the study of Sarlin et al. (2005) [34], it was observed that steeping during malting reduced the level of DON in non-treated samples, whereas the levels increased in artificially inoculated barley. There was a possibility that during steeping the level of DON can be sometimes decreased and is no longer detectable [20]. Through the first step of malting, steeping of barley grains apparently reduced *Fusarium* mycotoxin levels to below their quantification limits [32]. The highest DON and ZEN contents were detected in green malt, implying that the fungi had produced more mycotoxins during grain germination. Our investigation showed similar situation where after malting of untreated samples DON concentration was higher. This observation occurred after malting among less samples within inoculation treatment, whereas more samples showed a decrease of DON. Habler et al. (2016) [35] concluded that specific *Fusarium* species which contaminate the raw grain material might have different impacts on malt quality. In particular, the type B trichothecenes and *F. culmorum* DNA contents were increased dramatically, by up to 5400%, after kilning. By contrast, the concentrations of type A trichothecenes and *F. sporotrichioides* DNA decreased during the malting process. While the actual incidence of DON in barley was lower than that in wheat [36], a special attention needs to turn to wheat, because it is also one of the common raw materials used for beer production. Overall, malt generally contained ZEN in both treatments but not in unprocessed wheat and there was not any significant correlation between ZEN and DON in inoculated and control malt, but there was between ZEN and 3-ADON in inoculated treatment for malt. Similar findings for ZEN were obtained by Sarlin et al. (2005) [34], where production of ZEN in barley was followed during the malting process. Within grain from the control and inoculated treatment, only a few samples contained T-2 and HT-2 toxin in total. For barley, during four-year research, the percentage of positive T-2 and HT-2 samples was higher, ranging from 22% to 53%, with values between 26 and 787 µg kg^−1^ [37]. In our research, for T-2 and HT-2 toxins, there were substantial losses during malting. In 2014/2015, a total set of 25 samples of wheat grain and malt was analyzed for the content of mycotoxins DON, ZEN, and sum of T-2 and HT-2 toxins using the LC-MS/MS. Mycotoxin contamination data were significantly correlated with disease incidence and intensity, as well as with FCK. Of all mycotoxins, DON occurred most frequently. Still there were too many variations from which we can not conclude that disease parameters may also represent a specific indicator of mycotoxins prevalence in malt. The mycotoxin ZEN could be specific only for the wheat malt. In addition, in future, this research will be continued through investigation of the potential for formation and/or degradation of these mycotoxins during malting and brewing.

Our results have provided valuable information on the importance of selection of Fusarium resistant wheat varieties for malting industry. From a breeders standpoint, the tested wheat varieties ‘Srpanjka’, ‘Kraljica’, ‘Vulkan’ and ‘Os Alka’ may be recommended as a reliable material for cultivation for malting and brewing industry under heavy Fusarium pressure in this part of Europe.

## 4. Materials and Methods

### 4.1. Inoculum Production

Inoculum consisted of conidia of *F. culmorum* (IFA 104), DON chemotype and highly aggressive isolate, which was obtained from Institute of Biotechnology, IFA-Tulln, Austria. To produce macroconidia of *F. culmorum*, a mixture of wheat and oat grains (3:1 by volume) was soaked in water overnight in 250 mL glass bottles [38]. Water was decanted and seeds autoclaved. After seeding with the Fusarium strain, the seeds were kept for two weeks at 25 °C in the dark and thereafter incubated in the refrigerator for three weeks. Conidia were washed from the kernels and the concentration of the conidial suspension was set to 1 × 10^5^ mL^−1^ by haemocytometer. The spore suspension was set to a concentration so that single bottle contained a sufficient amount of suspension (>900 mL) which could be diluted in 100 L of water right before inoculation (100 mL per m^2^).

### 4.2. Field Trials

The field trial was set up at the Agricultural Institute Osijek (45°32′ N, 18°44′ E) where the soil type is eutric cambisol. The average annual precipitation in vegetation period during 2014/15 was 513 mm and the average annual temperature was 11.3 °C. The experimental plot area was 7.56 m^2^, where one treatment (control and artificially inoculated) was replicated in two plots. Twenty-five winter wheat varieties were artificially inoculated with *F. culmorum* during the flowering stage using tractor back-sprayer in the late afternoon and repeated two days later (Table 2). To maintain moisture at ears water was sprayed with tractor back-sprayer on several occasions during the day. Control group consisted of plants left to natural infection without usage of fungicide. For assessing disease attack, we calculated three parameters (general resistance, Type I resistance and fusarium colonized kernels (FCK)). The percentage of bleached spikelets (disease intensity) per plot was estimated according to a linear scale (0–100%) on day 22 after inoculation. FHB intensity per plot was taken as a measure for general resistance (GR). Disease incidence (percentage of diseased ears per plot, T1) was used as a measure for Type I resistance. The percentage of diseased heads was calculated after assessing a random sample of 30 heads on 22nd day after inoculation. For the determination percentage of FCK, 100 kernels of each variety were randomly selected and washed in 90% ethanol for 20 s. After evaporation of the alcohol, the seeds were placed on moist filter paper in Petri dishes and incubated at 25 °C at a relative air humidity of 80%. On the 6th day after incubation FCK was assessed. After harvest, the samples were collected for malting process and mycotoxin analysis.

### 4.3. Malting

The malting process started with drying the wheat grains to moisture content below 14% and then storing it around six weeks to overcome seed dormancy. Samples for malting weighted 200 g for each variety and were processed in an Automated Micro-malting Unit (Joe White Malting Systems, Perth, Australia) at Agricultural Institute Osijek. Samples were placed randomly within the micro-malting unit. The malting program consisted of a 37 h interrupted steep program (16 °C, 5 h submerged, 17 °C, 12 h air rest with 100% airflow, 17 °C, 6 h submerged, 18 °C, 12 h air rest with 100% airflow, 17 °C, 2 h submerged), a 96 h germination program (17 °C, 75% airflow, 1.5 turn every 2 h) and a 18 h kilning program (60 °C, 6 h; 65 °C, 3 h; 68 °C, 2 h; 70 °C, 2 h; 80 °C, 2 h; 83 °C, 2 h; 85 °C, 1 h). Rootlets were removed and the finished malt was then stored in plastic containers with caps at −20 °C until mycotoxin analysis.

### 4.4. Mycotoxin Analyses

Determination of DON, 3-ADON, HT-2, T-2, NIV, DAS, fusarenon X (FUS X) and ZEN with LC-MS/MS method in wheat grain and wheat malt was performed according to Ren et al. (2007) [39]: 10 g of wheat grain/malt was ground by IKA M20 (IKA, Staufen, Germany). The mycotoxins were extracted within 40 mL of 84:16 acetonitrile:water (*v*/*v*). The mixture was stirred for 3 min at high speed in a Waring LB10S blender (Waring, Stamford, CT, USA). The extract was filtered through Whatman No. 1 filter paper followed by glass microfiber filter (934-AH). Following filtration, a SPE column purification was performed by addition of 15 mL of extract to the SPE column (MultiSep 226 AflaZon+ Multifunctional Colums, RomerLabs, Tulln, Austria). After purification on SPE columns, an aliquot of 6 mL of clean-up solution was dried under gentle steam of high purity nitrogen (5.0) (Messer, Osijek, Croatia) at 50 °C. The residue was re-dissolved in 400 µL of mobile phase, transferred into vials and 20 µL was injected into LC-MS/MS (Sciex, Foter City, CA, USA). Perkin Elmer Series 2000 binary pump (PerkinElmer, Shelton, CT, USA) with auto sampler was combined with API 2000 Triple-quadrupole MS (SCIEX) for analysis. The Ascentis Express C-18 column was used for mycotoxin separation (150 × 2.1 mm; with 2.7 µm particle size). The column was heated to 45 °C, and the eluent A was 10 mM formic acid, and eluent B 10 mM of formic acid in methanol (pH of both eluents was set to 3.8 with ammonium hydroxide). The gradient started with 80% of eluent A, that was decreased to 50% within 10 min, followed to 20% in next 5 min, and to 0% until 16 min. The 100% B was held for 10 min, followed by equilibration to starting conditions for 4 min. Flow rate was set to 200 µL min^−1^. The MS/MS analysis was performed by using ESI source in both (positive and negative) modes, in two separate runs. The Ion Spray Voltage was set to −4500 V in negative and 5500 V in positive mode. Nitrogen was used as ion source and collision gas. Results were analyzed with Analyst software version 1.4.2 (Sciex, Foter City, CA, USA, 2008).

### 4.5. Method Validation

During validation of the method following parameters were included: extraction efficiency (*R_E_*), apparent recovery (*R_A_*), matrix effect (*SSE*) (signal suppression/enhancement), interlaboratory repeatability and reproducibility, limit of detection and limit of quantification, linearity (working range), and specificity in accordance with the EC 657/2002 [40]. The recovery experiments were performed on blank wheat samples (with no detected mycotoxins) by using 3 different concentrations (1×, 3×, and 10× LOQ) in triplicates. The matrix effect (enhancement or suppression), extraction efficiency and apparent recovery were assessed by comparing the average area of spiked samples with area of matrix-matched standard, and eluent diluted standard. The following Equations were used for the calculation according to Malachová et al., 2014 [40]:

(1) $$R_{E}\left(\%\right)=\frac{average\;area\;\left(spiked\;samples\right)}{average\;area\;\left(matrix\;matched\;standard\right)}\times 100$$

(2) $$R_{A}\left(\%\right)=\frac{average\;area\;\left(spiked\;samples\right)}{average\;area\;\left(eluent\;diluted\;standard\right)}\times 100$$

(3) $$SSE\left(\%\right)=\frac{average\;area\;\left(matrix\;matched\;standard\right)}{average\;area\;\left(eluent\;diluted\;standard\right)}\times 100$$

The repeatability was reported as relative standard deviation (RSD), calculated from spiked samples, while interlaboratoriy reproducibility was also calculated as RDS between three different days of analysis. For each analyte two specific transitions were monitored (qualifier and quantifier), and their ion ratio was also selected as additional confirmation point, with maximum tolerance up to 30% according to EC 657/2002 [41].

A seven-point calibration curve in methanol:water 50:50 was prepared from the stock solution (Mix 4 (A+B Trichothecenes & Zearalenone), Romer Labs, Tulln, Austria). Concentrations for each mycotoxin were approximately: 5, 10, 50, 100, 200, 500, and 1000 µL min^−1^ (the exact concentration may vary ±2% depending on the exact concentration in the purchased mix). For the quality control the calibration standard were passed every after every 25 samples including one quality control sample prepared by spiking of the plank wheat. Quantification was carried out by external calibration. The limit of detection (LOD) was defined as the concentration at which the signal to noise ratio equals to 3 and the limit of quantification (LOQ) was defined as the concentration where the signal to noise ratio equals to 10 by using the Analyst S-to-N script. All results were corrected for the apparent recovery, and recalculated according to the used clean-up. All validation parameters were summed up in the Table 3. Recovery was calculated using the wheat samples spiked with the mycotoxin mixed at three different levels with triplicate analyses conducted for each level.

### 4.6. Statistical Analysis

The values for Fusarium colonized kernels, disease intensity and incidence of individual assessment data was used. Correlation analyses were performed by Statistica 13 software (Version 13.3, TIBCO Software Inc., Palo Alto, CA, USA, 2017).

## Figures and Tables

**Table 1:** Correlation coefficients between DON and disease pa rameters in different treatments.

Trait	Control		Inoculum	
	Malt	Grain	Malt	Grain
FCK **	0.21	0.10	0.53 *	0.54 *
Intensity	0.05	0.00	0.39	0.38
Incidence	−0.06	0.01	0.42	0.55 *

* = significant at 0.01; FCK **—*Fusarium*-colonized kernels.

**Table 2:** Origin, year of release and susceptibility to Fusarium of 25 investigated winter wheat varieties.

Varieties	Origin *	Year of Release	Susceptibility ** to *Fusarium*
GOLUBICA	HR, AIO	1997	S
SUPER ZITARKA	HR, AIO	1997	S
BC ANICA	HR, BC	2010	S
SANA	HR, BC	1983	MS
ŽITARKA	HR, AIO	1985	MS
OS ALKA	HR, AIO	2003	MS
KATARINA	HR, AIO	2006	MS
BASTIDE	FRA	2003	MS
FELIX	HR, AIO	2007	MS
U1	HR, AIO	1936	MR
BEZOSTAYA-1	Former USSR	1955	MR
SRPANJKA	HR, AIO	1989	MR
FLAMURA 85	ROM	1989	MR
DIVANA	HR, JS	1995	MR
APACHE	FRA	1998	MR
LUCIJA	HR, AIO	2001	MR
DROPIA	ROM	2006	MR
RENATA	HR, AIO	2006	MR
OLIMPIJA	HR, AIO	2009	MR
VULKAN	HR, AIO	2009	MR
KRALJICA	HR, AIO	2010	MR
ANTONIJA	HR, AIO	2011	MR
SIRBAN PROLIFIC	HU	1905	R
RENAN	FRA	1991	R
GRAINDOR	FRA	2006	R

* AIO-Agricultural Institute Osijek, JS-Jost sjeme, BC-BC Institute; ** MR-moderately susceptible, S-susceptible, R-resistant.

**Table 3:** Validation parameters of the used LC-MS/MS method for mycotoxins quantification.

Analyte	Polarity	Retention Time (min)	*R_E_* (%)	*R_A_* (%)	*SSE* (%)	RSD Interday	RSD Intraday	LOD Matrix (ng g^−1^)	LOQ Matrix (ng g^−1^)
NIV	−	2.4	81	70	70	5.3%	9.6%	6.1	21.0
DON	−	2.5	102	101	99	6.0%	10.4%	4.8	15.1
FUS-X	+	2.6	72	64	89	2.8%	4.8%	2.7	9.8
3-ADON	+	3.1	92	88	96	5.1%	7.1%	5.15	16.2
DAS	+	14.9	84	97	117	7.9%	21.6%	2.1	7.1
HT-2	+	18.4	70	60	86	7.1%	14.0%	1.8	6.2
T-2	+	19.6	72	75	107	7.4%	12.8%	2.3	6.6
ZEN	−	19.8	73	82	109	11.2%	13.6%	1.2	3.1

*R_E_*—extraction efficiency; *R_A_*—apparent recovery; *SSE*—signal suppression/enhancement; RSD—relative standard deviation; LOD—limit of detection; LOQ—limit of quantification.
